# Using the consolidated framework for implementation research to guide a pilot of implementing an institution level patient informed consent process for clinical research at an outpatient setting

**DOI:** 10.1186/s40814-023-01234-0

**Published:** 2023-01-12

**Authors:** Xuling Lin, Joanne Yong Ern Yuen, Wei Quan Jeremy Chan, Tushar Gosavi Divakar, Nicole Chwee Har Keong, Lester Chee How Lee, Sumeet Kumar, Chew Seah Tan, Kim Chin Pauline Soon, Yee Pheng Amy Chew, Heriati Mohd Yazid, Farah Julieanna Mohd Saleh, Fenglong Cai, Fui Chih Chai, Nur Fakhirah Mohamed Azwan, Nurhidayah Mohamad Faizal, Siew Choo Lou, Siew Sin Priscilla Tan, Cut Marini Jarimin, Gowri Michael Stanley, Khadijah Hussien, Nurhazah Sanmwan, Nur Hidayah Amran, Nurliana Ramli, Shermyn Xiu Min Neo, Louis Chew Seng Tan, Eng King Tan, Elaine Lum

**Affiliations:** 1 Department of Neurology, National Neuroscience Institute, 11 Jalan Tan Tock Seng, Singapore, 308433 Singapore; 2 Department of Neurosurgery, National Neuroscience Institute, Singapore, Singapore; 3 Department of Neuroradiology, National Neuroscience Institute, Singapore, Singapore; 4 Specialist Outpatient Clinic Ambulatory Department, Singapore General Hospital, Singapore, Singapore; 5 Health Services & Systems Research, Duke-NUS Medical School, National University of Singapore, Singapore, Singapore

**Keywords:** Health resources, Implementation science, Informed consent, Health service research

## Abstract

**Background:**

In Singapore, research teams seek informed patient consent on an ad hoc basis for specific clinical studies and there is typically a role separation between operational and research staff. With the enactment of the Human Biomedical Research Act, there is increased emphasis on compliance with consent-taking processes and research documentation. To optimize resource use and facilitate long-term research sustainability at our institution, this study aimed to design and pilot an institution level informed consent workflow (the “intervention”) that is integrated with clinic operations.

**Methods:**

We used the Consolidated Framework for Implementation Research (CFIR) as the underpinning theoretical framework and conducted the study in three stages: Stage 1, CFIR constructs were used to systematically identify barriers and facilitators of intervention implementation, and a simple time-and-motion study of the patient journey was used to inform the design of the intervention; Stage 2, implementation strategies were selected and mapped to the Expert Recommendations for Implementing Change (ERIC) taxonomy; Stage 3, we piloted and adapted the implementation process at two outpatient clinics and evaluated implementation effectiveness through patient participation rates.

**Results:**

We identified 15 relevant CFIR constructs. Implementation strategies selected to address these constructs were targeted at three groups of stakeholders: institution leadership (develop relationships, involve executive boards, identify and prepare champions), clinic management team (develop relationships, identify and prepare champions, obtain support and commitment, educate stakeholders), and clinic operations staff (develop relationships, assess readiness, conduct training, cyclical tests of change, model and simulate change, capture and share local knowledge, obtain and use feedback). Time-and-motion study in clinics identified the pre-consultation timepoint as the most appropriate for the intervention. The implementation process was adapted according to clinic operations staff and service needs. At the conclusion of the pilot, 78.3% of eligible patients provided institution level informed consent via the integrated workflow implemented.

**Conclusions:**

Our findings support the feasibility of implementing an institution level informed consent workflow that integrates with service operations at the outpatient setting to optimize healthcare resources for research. The CFIR provided a useful framework to identify barriers and facilitators in the design of the intervention and its implementation process.

## Key messages regarding feasibility

- Research teams seek informed patient consent on an ad hoc basis for specific clinical studies, and there is typically a role separation between operational and research staff.
- Using the Consolidated Framework for Implementation Research (CFIR) as the underpinning theoretical framework, we identified intervention implementation barriers and facilitators and selected implementation strategies to pilot an institution level informed consent workflow that is integrated with clinic operations.
- Our findings support the feasibility of the intervention and could help inform the decision to adopt and adapt the intervention at resource limited healthcare settings to optimize resource use and facilitate long term research sustainability.

## Background

Clinical research is vital to the development of healthcare science. It lends understanding to disease development and trends; evaluates the safety, cost, and effectiveness of management strategies; and generates hypotheses for preclinical and translational drug development research. While much clinical research is appropriately conducted, instances of inappropriate research designs and misuse of data have been reported [1–3]. Such research misconduct can lead to misleading and potentially harmful findings and erode public trust in the healthcare and research community as a whole.

Singapore invests heavily in clinical research through various competitive national funding schemes via the National Research Foundation [4]. The current committed national budget for research, innovation, and enterprise from 2021 to 2025 (RIE2025) is valued at SGD$25 billion (USD$18.5 billion) [5]. In order to regulate the conduct of clinical research, Singapore enacted the Human Biomedical Research Act in 2015 (HBRA) [6]. On 1 November 2017, further human biomedical research regulations were established to govern the use of patient data [7]. With rare exceptions, patient consent is strictly required for their data to be accessed, collected, and analyzed for each research project. In addition, adherence to consent-taking guidelines and clear documentation of the consent-taking process for each patient are required [8]. While these regulations serve to uphold patient autonomy and to protect patient data and interest as top priorities, compliance to the HBRA demands investment of significant financial and human resources [9, 10].

Unlike established practices in many countries such as the USA where patients provide consent for their data to be used in research upon registration at hospital and/or as part of consent prior to investigations by operational staff [11], tertiary healthcare institutions in Singapore, active in clinical research, seek informed patient consent on an ad hoc basis for specific clinical studies [12]. In addition, there is often role separation between operational and research staff. Operational staff assist with administrative/clerical roles within the health service, while patient recruitment that includes obtaining informed consent for clinical studies, are conducted by research staff assisting specific Principal Investigators.

In response to the HBRA and with a view to facilitate future research processes in our institution, this study aimed to design and pilot an approach for an institution level informed consent for neuroscience clinical and health services research that is integrated with clinic operations. We hypothesized that clinic operations staff could be trained to facilitate informed consent if a workable process could be integrated into clinic operations. Findings are reported in line with the Standards for Reporting Implementation Studies (StaRI) [13].

## Methods

### Study objective

The objective of this implementation study is to demonstrate that the intervention, a process to obtain informed consent for clinical and health services research that is integrated with clinic operations at an institution level, is feasible. We defined feasibility as “the extent to which an intervention can be carried out in a particular setting or organization,” in line with the Implementation Outcomes Framework [14]. The overall goal of the intervention is to optimize resource alignment for research while ensuring compliance to the HBRA regulations and avoiding significant disruption to clinic operations.

### Theoretical framework

We used the Consolidated Framework for Implementation Research (CFIR) as the theoretical framework for this study [15, 16]. CFIR enables a systematic and comprehensive assessment of potential barriers and facilitators to implementation of an intervention, and it is well-used in health service research [16, 17]. The framework can be used (iteratively) at any stage of implementation—pre, during, post—to highlight specific factors that might affect successful implementation, influence the process, or attenuate the effectiveness of strategies. CFIR comprises 39 constructs and sub-constructs across 5 domains: intervention characteristics (key attributes of the intervention that may affect implementation success), outer setting (contextual factors such as external policies and incentives, patient needs and resources, inter-organizational networks, peer pressure), inner setting (structural characteristics, communications and networks, culture, etc.), characteristics of individuals (personal attributes of individuals affected including knowledge and beliefs about the intervention, self-efficacy, individual stage of change), and process (how to enact change) [16].

### Intervention

The intervention to be implemented is an *institution level informed consent workflow for clinical and health services research* targeted at patients attending neuroscience specialist outpatient clinics (two pilot sites) on their first visit. Institution level—meaning consent is sought not only for specific studies ad hoc for each Principal Investigator and study team (what currently happens), but for the use of patient data, captured during health service provision at an institution level, in current clinical and health service research involving the National Neuroscience Institute (single site or multi-institutional; local, regional, or international research); additional consent is sought for the use of data for future relevant institution review board (IRB)-approved studies. Importantly, providing institution level informed consent does not preclude patients from consenting to other studies; for example, when new data need to be collected to address specific research questions. The patient’s institution level informed consent would be documented manually and subsequently recorded in their electronic medical record.

### Study setting

#### The institution

The National Neuroscience Institute (NNI) is a Singapore specialist center for the management of patients with diseases of the central and peripheral nervous system, including stroke, dementia, epilepsy, Parkinson’s disease, brain tumor, and head injury. NNI provides neurology, neurosurgery, and neuroradiology inpatient and outpatient services as well as undergraduate and postgraduate education at two main campuses—Tan Tock Seng Hospital (TTSH) and Singapore General Hospital (SGH), and five partner hospitals—Changi General Hospital (CGH), Kandang Kerbau Women’s and Children’s Hospital (KKH), Khoo Teck Puat Hospital (KTPH), Sengkang General Hospital (SKH), and the upcoming Woodlands Health (WH). In addition to clinical services and education activities, NNI runs comprehensive neuroscience research programs that have received national and international accolades. Of approximately 100 NNI doctors (neurologists, neurosurgeons, and neuroradiologists), 50 conduct research studies as Principal Investigators and are supported by 40 clinical research staff and 4 research administrators at the two main campuses. In 2018 alone, NNI researchers were awarded eight Singapore National Medical Research Council (NMRC) grants and in 2019, the brain tumor research program was awarded nearly SGD$10 million (approximately USD $7.45 million) under the Open Fund—Large Collaborative Grant (OFLCG) to further translational research in glioblastoma [18].

#### The pilot sites

The neuroscience clinics located at the two main campuses, TTSH and SGH, were chosen as the pilot sites. Each of these two clinics serves approximately 2500–4500 patients per month and is supported by operations staff, nurses, and doctors. The majority of patients are referred from primary healthcare institutions/clinics while the rest are referred from the emergency department and other departments within the same hospital. For this report, the clinic at TTSH is labeled “Clinic A” and that at SGH “Clinic B.”

#### Overview of our process

The study was conducted from the perspective of the healthcare provider/institution and divided into three stages, each addressing one of the research questions below:

1. What are the constraints, barriers, and facilitators for the intervention?
2. What implementation strategies are needed to implement the intervention?
3. How effective are the implementation strategies?

##### Stage 1: Understand constraints, barriers, and facilitators

We (XL, TGD) completed an assessment using CFIR constructs to systematically identify potential constraints, barriers, and facilitators, taking into consideration day-to-day clinic operations (micro), institutional priorities (meso), and regulatory requirements of the HBRA (macro). The rationale was to surface critical constraints or barriers that should be addressed, as well as to inform selection and development of implementation strategies.

As part of understanding clinic constraints, we needed to identify possible points of the patient journey in which they could be approached to obtain institution level informed consent. To do this, we conducted a simple time-and-motion study. Two researchers (YEJY, WQJC) independently completed three observational visits over two typical days at each clinic: once during morning clinic sessions (9.00–11.00 am) and twice during afternoon clinic sessions (2.00–4.00 pm). Patient journeys from clinic registration to exiting the clinic were mapped for new patients (first presentation at clinic). Time taken at each station within the clinic was recorded. A total of 63 patients were observed. The roles of staff, including clinic administrative staff, nurse, physician, who interacted with the shadowed patients at each station were noted.

##### Stage 2: Select and develop implementation strategies

Findings from Stage 1 were used to inform the selection and development of implementation strategies. To facilitate consistent reporting, two researchers (XL and EL) worked together to map the selected strategies to the Expert Recommendations for Implementing Change (ERIC), a compilation of 73 discrete implementation strategies [19].

##### Stage 3: Pilot and adapt implementation process to encourage uptake of the intervention by staff and patients

We piloted and refined the implementation process in two sub-stages (3a and 3b).

- Stage 3a involved the clinical research coordinators (CRCs) and aimed to test the logistics of the implementation process.
- Stage 3b involved both the clinic operations staff and CRCs and aimed to examine the refined implementation process, including the collaboration and contribution by clinic operations staff and CRCs.

To minimize disruptions to clinic operations, the institution level informed consent was designed to be facilitated by clinic operations staff during patient registration. Patients with additional questions or who required special assistance would be attended to by the CRC after they have completed the registration for their clinic visit.

Although the research process is presented in a linear way, like most implementation studies, the process was iterative. For example, learnings from Stage 3a prompted adjustments to implementation strategies for Stage 3b. The implementation process was observed, analyzed, and adapted to improve adoption and to increase likelihood of sustainability of the intervention. We evaluated the effectiveness of the implementation strategies by the proportion of patients willing to provide informed consent and examined how adaptation influenced patient participation rates.

#### Research ethics

This study has been reviewed and approved by the SingHealth CIRB (CIRB No.: 2020/2456) and allows for the use of data for specific current neuroscience clinical and health services research. Additional IRB approval would still be required for the data to be used in future research.

## Results

### Stage 1: Understand constraints, barriers, and facilitators

The main constraints, barriers, and facilitators identified using CFIR are outlined below and summarized in Table 1.

**Table 1:** Summary of multilevel planning and intervention using the Consolidated Framework for Implementation Research (CFIR)

CFIR domain	CFIR constructs	Main constraints/barriers/challenges [facilitators]	Solutions	Implementation strategies^a^
Intervention characteristics	Relative advantage	[Compliance with HBRA without incurring additional cost]	Convey human and financial benefits of intervention to leadership and stakeholders	Develop stakeholder relationships; involve executive boards
	Complexity	Involvement of multiple stakeholders	Collaborate closely and gain their support	Develop stakeholder relationships; identify and prepare champions
	Trialability	Busy outpatient clinic; integration with clinic workflow to ensure smooth operations and patient safety	Understand clinic workflow; conduct pilot to test and refine	Conduct cyclical small tests of change; tailor strategies
	Adaptability	Different workflow and clinic cultures in both sites	Adjust implementation process to fit the different clinic environments	Promote adaptability; tailor strategies; conduct cyclical small tests of change
Outer setting	External policies and incentives	HBRA regulations caused an increase in resources required to support research	Align resources by integrating clinical and research efforts to recruit patients for clinical research	(This is the motivation for the intervention)
Inner setting	Structural characteristics	Minimal overlap in operations and manpower between research activities and clinical services	Design the intervention to allow optimization of resources	-
	Culture	Research is secondary to clinical service	Change management	Identify and prepare champions; train and educate stakeholders
	Implementation climate	Potential conflict with institution and clinic priorities	Convey human and financial benefits of intervention to leadership and stakeholders	Develop stakeholder relationships; involve executive boards
	Readiness for implementation	Potential negative impact due to structural characteristics, culture, and implementation climate	Address barriers from structural characteristics, culture, and implementation climate	Identify and prepare champions; train and educate stakeholders; develop stakeholder relationships
Characteristics of individuals	Knowledge and beliefs about the intervention	Inadequate knowledge of research and its importance amongst clinic operations staff	Staff training	Develop educational materials; make training dynamic; conduct ongoing training; provide assistance
	Self-efficacy	Need to assure clinic operations staff of their abilities to carry out the intervention	Role play during training sessions to build up confidence; CRCs to work alongside clinic operations staff to emphasize dynamic work-sharing	Conduct ongoing training; make training dynamic; provide assistance; model and simulate change
Process	Planning	Novel and complex nature of the intervention, and the importance of seamless integration with clinic processes	Careful planning before implementation together with stakeholders involved and institution leadership	Develop stakeholder relationships; involve executive boards
	Engaging	Need for appropriate personnel to lead, manage, and champion the intervention	Appoint committee with two co-leaders to govern and lead the implementation; engage CRCs and senior clinic operations staff to champion the intervention	Develop stakeholder relationships; involve executive boards; identify and prepare champions; obtain formal commitments
	Executing	Feasibility of pre-consultation intervention and dynamic collaboration between clinic operations staff and CRC	Conduct pilot in two stages to examine the feasibility of pre-consultation intervention and dynamic collaboration separately	Assess for readiness and identify barriers and facilitators; conduct cyclical small tests of change; purposefully examine the implementation
	Reflecting and evaluating	Morale of staff and long-term sustainability	Positive publicity and talks; gather feedback and make appropriate adjustments to workflow	Model and simulate change; capture and share local knowledge; obtain and use feedback

^a^Implementation strategies mapped to the Expert Recommendations for Implementing Change (ERIC) [19]

#### Intervention characteristics

The intervention was perceived to have relative advantage over the status quo, in that it had the potential to facilitate future research in line with HBRA requirements without incurring significant investment. The implementation of the intervention was considered complex due to interdependent processes that required close collaboration between multiple parties: the clinical research coordinators (CRCs), operations staff, clinical team, and clinic management team. Trialability was important to enable integration and ownership of the intervention and implementation process by clinic operations staff, CRCs, and the clinical team. We anticipated that the institution level informed consent would need to be conducted within a constrained time window in clinic.

#### Outer setting

The outer setting describes the external economic, political, and social context of an organization and has the ability to influence an intervention implementation. In this context, the HBRA was the main external policy that drove the need for this intervention. Since 1 November 2017, HBRA mandates that patient consent, with rare exceptions, is required for their data to be used for research project and dictates strict guidelines involving consent-taking processes and documentations for each patient [8]. With these regulations, the HBRA has directly curtailed clinical research in resource-limited settings and indirectly influenced research culture in hospitals. Specifically, the growing emphasis on HBRA compliance has resulted in a significant amount of funds and resources being spent on patient recruitment and data management each year. Ensuring HBRA compliance, while desirable, has led to a culture of (over-)compliance reflected in foundational practices and expectations amongst research and research administrative staff. As such, each project employs at least one CRC to assist with HBRA compliance resulting in multiple CRCs conducting patient recruitment in clinic for various projects. From a systems point of view, such arrangements adopted in response to HBRA generate significant production-level waste, which may be addressed by and perpetuates the need for this institution level intervention.

#### Inner setting

The proposed intervention conflicted with traditional institution and clinic priorities, raising concerns under the constructs of “Structural Characteristics,” “Culture,” and “Implementation Climate,” which would spill over to “Readiness for Implementation.”

Traditionally, there was a dichotomy between service delivery and research. The mission of the neuroscience clinic was to provide outpatient clinical services. Clinic operations and key performance indicators were structurally geared towards improving service efficiency and optimizing patient safety, while research activities were led by neurologists or neurosurgeons and supported by grant-funded research assistants. This separation between clinical services and research nurtured and reinforced a mindset and culture among institution leadership and staff—research was considered a non-core activity to be undertaken with additional human and financial resources. Given these structural characteristics and the prevailing culture, a less receptive climate for the intervention was possible.

On the other hand, the institution had many established clinicians and leaders active in clinical research who would welcome optimal utilization of research resources. Our intervention, if successfully implemented, could support a large number of clinical studies within the institution. The anticipated reduction in human resources and potential cost-savings for research at an institution level are likely to garner support from the institution’s leadership.

#### Characteristics of individuals

We examined the characteristics of clinic operations staff co-opted for the implementation to gauge capability and readiness for change. The traditional delineation of clinical service and research activities meant that clinic operations staff were neither equipped with adequate knowledge about conducting research, nor understood the value of research. This was an important barrier to address which would have an impact on staff self-efficacy in facilitating institution level informed consent.

#### Process

To increase the likelihood for support, success, scalability, and sustainability of the intervention, we engaged with stakeholders at multiple levels to plan and execute the implementation—the institution leadership, clinical management team, and clinic operations staff.

#### Clinic workflow and patient journey

**Fig. 1:**
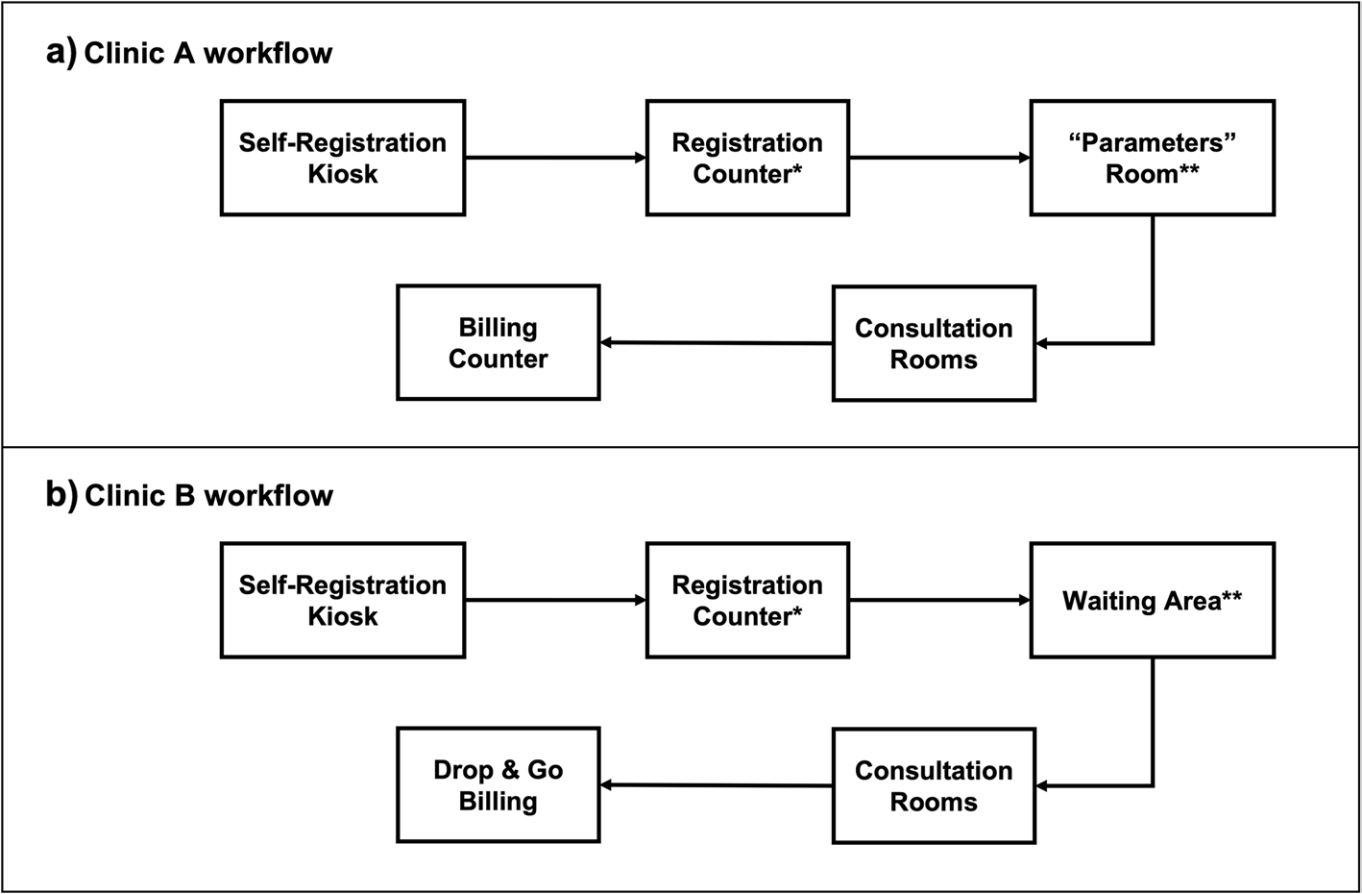
Patient journey in Clinic A and Clinic B. Self-registration kiosk: All patients would be directed to the self-registration kiosk upon arrival at the clinic to obtain a queue chit that has a unique queue number with/without their assigned consultation rooms. Registration counter: All first visit patients would not have their assigned consultation rooms displayed on the queue chit obtained at the self-registration kiosk and would have to be registered manually at the registration counter. “Parameters room”: All first visit patients at Clinic A would be directed to this room for height, weight, and blood pressure measurements before consultation with the doctor. Waiting area: First visit patients at Clinic B would proceed to the waiting area directly after registration. Consultation rooms: Consultation with the doctor would be held in assigned consultation rooms as reflected on the queue chit (time spent not captured). Billing counter: Patients in clinic A would be directed to the billing counter for payment. Drop & go billing: Patients in Clinic B would be given the option for drop & go billing and have their bills mailed to them for subsequent payment electronically or via AXS stations. *Clinic operations staff would approach the first visit patients for consent-taking at the registration counter. **CRCs or clinic nurses would approach the first visit patients for consent-taking at the “parameters” room (Clinic A) and waiting area (Clinic B)

At Clinic A (Fig. 1a), all first-visit patients would present at the registration counter before being directed to a “parameters” room for height, weight, and blood pressure measurements. Subsequently, patients were directed to seats outside their respective doctor’s rooms to await clinical consultation. Post-consultation, patients were directed to a common area in front of the billing counters to await their turn to make payment. In contrast to Clinic A, patients who visited Clinic B (Fig. 1b) were directed to a common area to await clinical consultation after registration. Post-consultation, the patients were directed to return home as their bill would be sent via text-messaging or mail.

The average time taken at each station is presented in Table 2. Overall, patients spent an average of 8.65 and 2.40 min at the stations in Clinics A and B, respectively. Time spent in clinical consultation was excluded as this varied depending on the needs of the patient and was not the target station for informed consent. In both clinics, the clinical consultation is a known bottleneck for patient flow identified through internal clinic audits (unpublished internal reports).

**Table 2:** Average time taken per station in clinic

	Average time taken, minutes (range)		
	Registration counter	“Parameters” room	Billing counter
Clinic A	2.50 (1.23–4.75)^a^	2.40 (1.48–5.25)^b^	3.75 (2.17–9.15)^c^
Clinic B	2.40 (0.34–6.83)^d^	Not applicable^e^	Not applicable^f^

^a^Number of patients tracked = 11

^b^Number of patients tracked = 12

^c^Number of patients tracked = 15

^d^Number of patients tracked = 25

^e^Clinic B does not have this station

^f^Clinic B sends the bill to patients

Given these findings, we identified pre-consultation as the most appropriate part of the patient journey for informed consent at both clinics as it conferred several advantages: (a) it does not contribute to clinic congestion, (b) it would not affect patients’ overall time spent in clinic, and (c) should consent-taking be interrupted, it can resume post-consultation. In contrast, conducting informed consent towards the end of the patient journey (billing) was deemed more challenging as (a) it would be difficult to identify first-visit patients at the billing counter, and (b) there will be considerable loss of potential participants due to the increasing number of patients choosing to use the drop-and-go billing option at Clinic B. Specifically, the drop-and-go billing method allows patients to leave the clinic right after clinic consultation and have their bills mailed to them for subsequent payment electronically or via AXS stations, interactive 24-h self-service terminals located at over 700 sites Singapore-wide, and selected convenience stores. Hence, for Clinic A, institution level informed consent would be conducted at either the registration counter or before entering the “parameters” station, while for Clinic B, the opportunity to approach patients for informed consent would be at registration and prior to clinical consultation.

### Stage 2: Select and develop implementation strategies

Implementation strategies were targeted at three groups of stakeholders: institution leadership, clinic management team, and clinic operations staff. The implementation strategies selected to address constraints and barriers identified, or to leverage facilitators, are summarized in Table 1 and outlined as follows.

Critical to the success of the implementation were buy-ins from institutional leadership and stakeholder engagement. We first sought support from institution leadership by clearly defining the potential benefits of the intervention—resource alignment and cost-savings within the context of HBRA regulations. The institution leadership took time to scrutinize the proposed intervention and implementation plans and subsequently agreed to render support. A committee of five, comprising a representative from neurology (XL, TGD) and neurosurgery (NCHK, LCHL) at each campus and neuroradiology (SK - at TTSH campus), was appointed to lead the implementation; the committee in turn reported directly to the NNI research director. The committee laid out the data governance structure which consisted of a workflow for data management and data release, as well as publication guidelines for NNI researchers. Additionally, two co-leaders (XL, TGD) from the committee were chosen to facilitate the day-to-day implementation at each clinic, and were responsible for obtaining IRB approval for the study.

Next, we engaged the clinic management teams with a clear vision and mission for the intervention and proactively discussed the expected challenges during implementation. The clinic management teams identified several more experienced clinic operations staff to spearhead the initiative. We negotiated with clinic management teams for the identified operations staff to undergo training sessions, and to subsequently roster those who have undergone training at the registration counters during the pilot.

For clinic operations staff, provision of structured training was an important implementation strategy. We developed training materials to prepare clinic operations staff with the necessary knowledge to conduct research activities and raise their understanding of research, the value of research, the rationale, and importance of the proposed intervention, and skills to conduct the intervention. Training sessions also provided an opportunity to address any misconceptions or questions regarding the intervention. We conducted two 1–2 h face-to-face training sessions with each of the clinic staff approximately 2–4 weeks before their participation in the pilot. The training sessions included role plays to further build their confidence and self-efficacy in consent-taking. At the end of their training, the staff had to complete online learning modules and get Collaborative Institution Training Initiative (CITI) and SingHealth HBRA e-training certified.

The two-stage pilot provided an opportunity for clinic operations staff to observe CRCs conduct the informed consent in Stage 3a, prior to being involved in Stage 3b. We recognized that not all staff would be ready for change within the same duration. For those who did not feel ready, we provided further support and delayed their participation in the pilot. At 3 months post-pilot, all clinic operations staff who had undergone the research training were actively participating in the intervention.

Ongoing communications to increase or maintain visibility of the intervention is necessary to sustain staff motivation. We celebrated each implementation milestone and participation of clinic operations staff via articles in the NNI newsletter distributed to all staff in the organization. Roadshows within the institution were planned and used to garner support for the intervention and also to recruit more outpatient clinics in preparation for scale-up.

### Stage 3: Pilot and adapt implementation process to encourage uptake of the intervention by staff and patients

The intervention was executed in two stages over a 2-month period at each clinic—Stage 3a and 3b for the first and second month, respectively. The 2-month period at TTSH was from 8 October 2020 to 8 December 2020, and SGH from 5 November 2020 to 31 December 2020. At each stage, the team gathered to discuss and propose changes to improve the existing processes and tested changes using the Plan-Do-Study-Act (PDSA) cycles [20].

### Stage 3a

The intervention was operationalized by CRCs at Clinics A and B. The CRCs were stationed at the registration counter of each clinic, and first-time patients were approached for institution level informed consent. Where patients were observed to experience cognitive challenges in providing signed consent, the CRC informed and coordinated with the clinical team to assess if this was so, and if appropriate, to certify the patient unfit for providing institution level informed consent. Patients were determined to have cognitive challenges when they expressed difficulties in understanding or appreciating information, making reasoned choices and/or expressing their decisions. The patients and their legal proxies and/or caregivers were explicitly informed that the certification for inability to sign consent was specific for the purpose of this study. For patients who were certified to have no mental capacity to provide consent, informed consent was sought from their legal proxies.

In Stage 3a, a total of 444 first-time patients were approached at pre-consultation in Clinics A and B. Of these, 78.5% (234/298) and 72.6% (106/146) from Clinic A and B, respectively, agreed to provide informed consent. Overall, 76.6% (340/444) of first-time patients approached provided informed consent, indicating the feasibility of conducting the institution level informed consent at pre-consultation.

Nevertheless, the team identified two implementation barriers: (a) the need for a translator and a witness to be present for signed consent for patients who did not speak or understand English and (b) the need to modify current clinic protocol which required clinic operations staff at Clinic A to contact respective doctors to seek approval before proceeding with patient registration for those who turned up late for their appointment. These were addressed through adjustment of the intervention at Stage 3b.

### Stage 3b

The Stage 3b phase was jointly operationalized by two teams consisting of clinic operations staff and CRCs at Clinics A and B.

Given the challenges identified in Stage 3a, we engaged a professional third-party translation service to provide a Mandarin version of the institution level informed consent form. This reduced the need for a translator and witness when recruiting Mandarin-speaking patients, which formed a substantial proportion of our patients. Discussions with clinic management and clinical teams resulted in modification of clinic protocol at Clinic A—clinic operations staff would need to seek approval for patient registration only if the patient was more than 30 minutes late and came during the last hour of the respective doctor’s clinic session. These refinements not only avoided delays at the registration counter, but also provided clinic operations staff with greater autonomy, further enabling them to facilitate institution level informed consent.

In Stage 3b, a total of 810 first-time patients were approached at pre-consultation in Clinics A and B. Of these, 81.5% (517/634) and 66.5% (117/176) from Clinic A and B, respectively, agreed to provide informed consent. Overall, 78.3% (634/810) of first-time patients approached provided informed consent. The refinements made following Stage 3a resulted in an increase in participation rates at Clinic A, but not Clinic B and could be related to the lack of non-Mandarin speaking clinic operations staff at Clinic B who would not utilize the Mandarin version of the institution level informed consent form. In addition, the operational changes in workflow for patients who turned up late for appointments was only relevant to Clinic A.

Overall, for the pilot, we found that the implementation of the intervention needed to be responsive to clinic service needs. For example, when the clinic registration counter was congested with patients, we modified the workflow to allow clinic operations staff to focus on patient registration, leaving the CRCs to conduct the informed consent with eligible patients. Different management structures and staff rostering in each clinic meant that we adapted the timing and duration of the training sessions to minimize disruptions to existing systems. Specifically, for Clinic A, we coordinated half day training sessions according to the combined schedule of the operations staff; for Clinic B, there was a preference to attend training during lunch time and we reduced the duration but increased the number of training sessions. Making these adjustments contributed to greater acceptance of the intervention among clinic operations staff and management teams.

## Discussion

This intervention, to the best of our knowledge, is the first of its kind in Singapore. The institution level informed consent workflow was successfully piloted at two busy neuroscience outpatient clinics, lending support to plans for adaptation and scale-up across the institution including all outpatient clinics as well as inpatient admissions in NNI. This intervention resolves several research challenges for our institution and may be useful for other settings.

First, our intervention integrated clinical service and research efforts to support institution level clinical research in a way that complies with HBRA requirements. Such resource alignment is critical for long-term sustainability of research in the institution.

Second, the institution level informed consent workflow reopens the possibility of conducting retrospective studies and the study of disease trends. The current regulations demand the foresight of clinical researchers to design specific studies and obtain informed consent from patients for each study. For rare and emerging, especially deadly diseases, this reduced access to data would make retrospective analysis and reporting almost impossible.

Third, the institution level informed consent workflow was implemented without unduly lengthening the patient journey. Consent-taking and documentation for research purposes typically take up a significant amount of time. This competes with time needed for clinical management of patients, which for outpatient consultations are already truncated to between 10 and 15 min per patient.

Fourth, co-opting clinic operations staff to facilitate the institution level informed consent was a healthcare manpower-sparing strategy, in terms of both cost and availability. Healthcare staff are costly to deploy, rostering is necessarily lean, and rightly prioritizes clinical rather than research duties. Often, clinicians are required to seek external sources of funding to hire manpower and purchase required resources for research. Funding challenges are amplified for clinicians in rarer and/or smaller subspecialities where it is harder to secure consistent research funding.

Finally, the CFIR was useful for planning, iterative refinement of strategies, and implementation of the intervention. Notably, the study team did not receive any complaints from patients with regards to being approached for institution level informed consent at registration/pre-consultation or about the consent-taking process. This may be a signal that the intervention is sufficiently integrated with clinic processes.

We found no similar precedent for obtaining institution level informed consent in an outpatient setting. As such, we offer our study as a useful baseline which contributes to the literature. While not directly transferable to other hospitals or countries due to contextual differences, our findings nevertheless provide a starting point for other institutions to consider similar interventions, adjusted to local research regulations.

### Lessons learned

Consistent with organizational change literature, most of the resistance towards the intervention and implementation arose due to internal factors such as established rules, regulations, internal policies, and culture of the organization [21, 22]. To ensure successful implementation, we worked within these constraints while carefully pushing these boundaries through collaboration, providing support, building working relationships, and persuasion, to create change over time [23–25]. During the pilot, we acknowledged that changing “the way we do things” is hard and were mindful not to rush clinic operations staff who did not feel ready to facilitate informed consent. We believe that supporting staff in empowering ways resulted in their willingness to be involved.

Despite the successful implementation, recent local developments of the COVID-19 pandemic affecting TTSH have greatly impacted continuing efforts [26]. Restrictions were introduced by the institution in line with the Singapore Ministry of Health which barred research staff from clinical areas. As a result, we observed inconsistent facilitation of institution level informed consent by clinic operations staff due to additional administrative duties, coupled with research staff being unable to lend support. However, we note that despite the pandemic, participation rates remained largely consistent, which suggests that the implementation process is robust, adaptable, and functional despite external threats.

### Strengths and limitations

The findings generated from this study have demonstrated the feasibility of implementing an intervention of this nature on a larger scale. While the intervention was developed in response to the HBRA in Singapore, it remains relevant to academic centers worldwide that are regulated by ethical frameworks and necessitate patients’ consent prior to use of their health data for research. The use of CFIR as the underpinning theoretical framework allows transferability of the intervention to other settings. Limitations of the current study are that only quantitative data was collected to determine feasibility of the intervention, the cost of implementation was not quantified, and patients were not integrated as research partners when planning the pilot study.

Moving forward, a qualitative component would be useful for understanding the perspectives of stakeholders, clinic operations staff, and patients with regard to the intervention, its usefulness for facilitating future research, and suggestions for improvement. We will seek patients/patient advocates as research partners to provide input at the planning and design stage of our next study: a qualitative survey among patients who declined participation in the informed consent to systematically collect their reasons for rejection. We believe that the results from this survey will allow us to better understand our local patients’ reluctance to participate in clinical research, devise strategies to address their concerns, and thereby improve future research participation rates. Subsequently, we hope to conduct surveys on patient reported experience measures among all first visit patients in clinic and aim to compare and evaluate the impact of patients’ experience on their decision for participation in clinical research.

## Conclusion

In the ever-changing environment of neuroscience, research plays a key role in understanding disease trends and developing management methods to help improve the lives of patients. However, with stricter regulations placed upon research, data collection required greater resources, resulting in the need for an innovative solution to help reduce waste, and invigorate the research environment. Institution level informed consent embedded within clinic operations is a feasible way to optimize research resources. The CFIR served as a useful framework to identify and understand key barriers and facilitators in the development of the intervention and its implementation process.

## Data Availability

Available.
